# Major differences in clinical presentation, diagnosis and management of men and women with autosomal inherited bleeding disorders

**DOI:** 10.1016/j.eclinm.2021.100726

**Published:** 2021-01-29

**Authors:** F. Atiq, J.L. Saes, M.C. Punt, K.P.M. van Galen, R.E.G. Schutgens, K. Meijer, M.H. Cnossen, B.A.P. Laros-Van Gorkom, M. Peters, L. Nieuwenhuizen, M.J.H.A. Kruip, J. de Meris, J.G. van der Bom, F.J.M. van der Meer, K. Fijnvandraat, I.C. Kruis, W.L. van Heerde, H.C.J. Eikenboom, Frank W.G. Leebeek, S.E.M. Schols, For the WiN, RBiN and TiN study groups

**Affiliations:** a Department of Hematology, Erasmus MC, University Medical Center Rotterdam, Rotterdam, the Netherlands; b Hemophilia Treatment Center Nijmegen-Eindhoven-Maastricht, the Netherlands; c Department of Hematology, Radboud University Medical Center, Nijmegen, the Netherlands; d Benign Hematology Center, Van Creveldkliniek, University Medical Center Utrecht and University Utrecht, Utrecht, the Netherlands; e Department of Hematology, University Medical Center Groningen, Groningen, the Netherlands; f Department of Pediatric Hematology, Erasmus MC-Sophia Children's Hospital, University Medical Center Rotterdam, Rotterdam, the Netherlands; g Emma Children's Hospital, Amsterdam UMC, University of Amsterdam, Pediatric-Hematology, Amsterdam, the Netherlands; h Department of Hematology, Maxima Medical Center Eindhoven, Eindhoven, the Netherlands; i Netherlands Hemophilia Society, Leiden, the Netherlands; j Department of Clinical Epidemiology, Leiden University Medical Center, Leiden, the Netherlands; k Jon J van Rood Center for Clinical Transfusion Medicine, Sanquin Research, Leiden, the Netherlands; l Department of Internal Medicine, Division of Thrombosis and Hemostasis, Leiden University Medical Center, Leiden, the Netherlands; m Department of Plasma Proteins, Sanquin Research, Amsterdam, the Netherlands; n Enzyre BV, Novio Tech Campus, Nijmegen, the Netherlands; o Einthoven Laboratory for Vascular and Regenerative Medicine, Leiden University Medical Center, Leiden, the Netherlands

**Keywords:** Hemorrhagic disorders, Inherited coagulation disorders, Sex characteristics, von Willebrand disease, Blood platelet disorders, Hemorrhage

## Abstract

**Background:**

In recent years, more awareness is raised about sex-specific dilemmas in inherited bleeding disorders. However, no large studies have been performed to assess differences in diagnosis, bleeding phenotype and management of men and women with bleeding disorders. Therefore, we investigated sex differences in a large cohort of well-defined patients with autosomal inherited bleeding disorders (von Willebrand disease (VWD), rare bleeding disorders (RBDs) and congenital platelet defects (CPDs)).

**Methods:**

We included patients from three nationwide cross-sectional studies on VWD, RBDs and CPDs in the Netherlands, respectively the WiN, RBiN and TiN study. In all studies a bleeding score (BS) was obtained, and patients filled in an extensive questionnaire on the management and burden of their disorder.

**Findings:**

We included 1092 patients (834 VWD; 196 RBD; 62 CPD), of whom 665 (60.9%) were women. Women were more often referred because of a bleeding diathesis than men (47.9% vs 36.6%, *p* = 0.002). Age of first bleeding was similar between men and women, respectively 8.9 ± 13.6 (mean ±sd) years and 10.6 ± 11.3 years (*p* = 0.075). However, the diagnostic delay, which was defined as time from first bleeding to diagnosis, was longer in women (11.6 ± 16.4 years) than men (7.7 ± 16.6 years, *p* = 0.002). Similar results were found when patients referred for bleeding were analyzed separately. Of women aging 12 years or older, 469 (77.1%) had received treatment because of sex-specific bleeding.

**Interpretation:**

Women with autosomal inherited bleeding disorders are more often referred for bleeding, have a longer diagnostic delay, and often require treatment because of sex-specific bleeding.

**Funding:**

The WiN study was supported (in part) by research funding from the Dutch Hemophilia Foundation (Stichting Haemophilia), Shire (Takeda), and CSL Behring (unrestricted grant).

**Research in context**

**Evidence before this study**

In recent years, more awareness is raised about sex-specific dilemmas in inherited bleeding disorders. Heavy menstrual bleeding is often a presenting symptom of a bleeding disorder. We have previously reported that 81% of all women with von Willebrand disease experienced menorrhagia, whereas over 50% of women experienced bleeding complications after childbirth or pregnancy loss.

**Added value of this study**

This is the first study to investigate sex differences in a large cohort of well-defined patients with different types of autosomal inherited bleeding disorders. Women are more often referred to the hospital because of bleeding episodes, and more often require treatment because of sex-specific bleeding compared to men. Although the age of first bleeding was similar between men and women with autosomal inherited bleeding disorders, the diagnostic delay was on average 6 years longer in women.

**Implications of all the available evidence**

There are important differences in presentation, diagnosis and management of men and women with autosomal inherited bleeding disorders. When the diagnosis of a bleeding disorder is delayed, women are at risk for not receiving appropriate treatment in case of bleeding or prophylaxis for surgery, dental procedures or during child delivery. Physicians should be more aware of bleeding disorders in women who present with a personal bleeding diathesis, and should investigate them earlier for bleeding disorders.

Alt-text: Unlabelled box

## 1 Introduction

Most studies on hereditary bleeding disorders have historically focused on hemophilia A and B, both X-linked disorders mainly affecting men. The vast majority of other bleeding disorders are autosomal inherited, and therefore sex differences may have an important impact on diagnosis, clinical characteristics and management of patients.

Autosomal inherited bleeding disorders predominantly consist of patients with von Willebrand disease (VWD), followed by patients with a rare hereditary bleeding disorder (RBD) which encompass a heterogeneous group of rare coagulation factor deficiencies and disorders of the fibrinolytic system. Lastly, hereditary platelet disorders (CPDs) are autosomal inherited bleeding disorders of primary hemostasis, affecting both men and women.

VWD, the most common inherited bleeding disorder, is characterized by mucocutaneous bleeding such as heavy menstrual bleeding (HMB), epistaxis and gum bleeds [1]. VWD is divided into three types, based on the quantitative or qualitative defect of von Willebrand factor (VWF) [1]. Type 1 VWD, which accounts for 70–80% of VWD patients, is characterized by quantitatively reduced VWF levels [2]. Type 2 VWD, which affects about 20% of VWD patients, is characterized by an abnormal function of VWF. Type 3 VWD, the most severe form of VWD, affecting less than 5% of patients, is characterized by the absence of VWF [1,3]. Type 1 and 2 VWD usually have an autosomal dominant inheritance pattern, whereas type 3 VWD is an autosomal recessive disorder.

RBDs are most often inherited as autosomal recessive disorders, except for dysfibrinogenemia and some cases of FXI deficiency, which are inherited autosomal dominant [4]. Rare coagulation factor deficiencies refer to deficiencies of fibrinogen, factor (F) II, FV, combined FV&FVIII, FVII, FX, FXI and FXIII. Patients with a rare coagulation factor deficiency have a diverse clinical presentation. In addition, there is a poor correlation between factor activity levels and bleeding phenotype [5]. Disorders of fibrinolysis consist of deficiencies of plasminogen activator inhibitor type 1 or α2-antiplasmin, or unspecified hyperfibrinolysis. The bleeding phenotype of patients with a disorder of fibrinolysis is characterized by delayed bleeding after trauma and interventions and bleeding in tissues with high fibrinolytic activity, such as HMB and epistaxis [6].

CPDs are disorders of primary hemostasis caused by defects in adhesion, activation, secretion, or aggregation of platelets [7]. Patients typically present with mucocutaneous bleeds or persistent bleeding following a hemostatic challenge such as dental extraction, invasive procedures or childbirth [8].

Although the autosomal inheritance pattern of these bleeding disorders should lead to an equal distribution amongst men and women, differences in prevalence have been reported [1,9,10]. Moreover, increasingly more awareness is raised in recent years about sex-specific problems in patients with inherited bleeding disorders due to menstruation and delivery as physiologic hemostatic challenges in women [11,12]. To our knowledge, no comprehensive studies have been conducted yet to investigate sex differences in symptoms, diagnosis and management of patients with autosomal inherited bleeding disorders.

Therefore, we investigated sex-specific differences in clinical phenotype, diagnosis and management of men and women with autosomal inherited bleeding disorders in a large cohort of well-defined patients with VWD, RBDs and CPDs from three nationwide cross-sectional studies in the Netherlands.

## 2 Methods

### 2.1 Nationwide studies on VWD, RBD and CPD patients

For this study, we included patients from three nationwide cross-sectional studies on VWD, RBDs and CPDs in the Netherlands, respectively the Willebrand in Netherlands (WiN) study, Rare Bleeding disorders in Netherlands (RBiN) study, and Thrombocytopathy in the Netherlands (TiN) study. The WiN study was performed between 2007 and 2009 and included 834 VWD patients [13,14]. The RBiN study was performed between 2017 and 2019 and included 263 patients with RBDs [15]. The TiN study was performed between 2016 and 2018 and included 62 patients with confirmed CPDs [16]. These three studies included patients from all Hemophilia Treatment Centers in the Netherlands. All studies were performed according to the Declaration of Helsinki and approved by the Medical Ethical Committees of all participating centers. All participants signed informed consent. This manuscript was written in adherence to the STROBE guidelines.

### 2.2 Inclusion criteria

The inclusion criteria for the WiN study were hemorrhagic symptoms or a family history of VWD, and historically lowest VWF antigen level (VWF:Ag) and/or VWF activity (VWF:Ab) and/or VWF collagen binding activity (VWF:CB) ≤ 0.30 IU/mL and/or factor VIII activity (FVIII:C) ≤ 0.40 IU/mL (for type 2N VWD) [13,17,18]. Type 1 VWD was categorized as VWF:Ab/VWF:Ag ratio >0.6, whereas type 2 VWD was categorized as VWF:Ab/VWF:Ag ratio ≤0.6 [3]. Type 3 VWD was defined as VWF activity and antigen levels ≤0.05 IU/mL and VWF propeptide ≤0.05 U/mL [3]. The inclusion criteria for the RBiN study were a coagulation factor level below the lower limit of normal, and/or a proven heterozygous, compound heterozygous or homozygous mutation for the rare coagulation factor deficiencies and α2-antiplasmin deficiency. Patients with a PAI-1 deficiency were eligible for inclusion if the PAI-1 activity was below the detection limit of the assay and the PAI-1 antigen level was below the lower limit of normal (reference range 3.4–39 ng/ml). Patients with hyperfibrinolysis were eligible if the euglobulin clot lysis ratio before and after application of a tourniquet was ≥5.8 (reference range 1.2–5.7, locally validated assay). From the TiN study, we included patients with a confirmed CPD: Bernard Soulier Syndrome, Glanzmann thrombasthenia, Adenosine diphosphate (ADP) pathway defect, Thromboxane A2 (TxA2) pathway defect and dense granule deficiency [16].

### 2.3 Assessment methods

At inclusion in the studies, patients filled in an extensive questionnaire containing questions on age of first bleeding and treatment, reason for referral, age of diagnosis and bleeding requiring hemostatic treatment in the year prior to inclusion in the study. Additionally, in the WiN study a self-administered Tosetto bleeding score (BS) was obtained, whereas in the RBiN and TIN studies the ISTH-BAT was obtained by the investigator [15,19,20]. We have previously shown that the self-administered BS obtained in the WiN study was comparable to the investigator obtained BS [14]. Lastly, blood samples were drawn to measure relevant coagulation factor levels in a central laboratory for each of the studies [13,15,18,21,22]. Laboratory measurements have been described in detail previously [13–16].

### 2.4 Definitions

Diagnostic delay was defined as age of diagnosis minus age of first bleeding. To make a better comparison between men and women, sex-specific bleeding was excluded from BS. In VWD, BS without sex-specific bleeding was defined as total BS minus the BS item for HMB, postpartum hemorrhage (PPH) and circumcision. In patients with RBDs and CPDs, BS without sex-specific bleeding was defined as the ISTH-BAT minus BS items for HMB, PPH, ovulation bleeding and bleeding during circumcision [19]. These definitions differ due to the fact that in the Tosetto BS, ovulation bleeding is not an individual item [20]. To determine the proportion of women experiencing HMB, only women ≥12 years of age were taken into account. For PPH, only women who had at least one delivery were taken into account. Both these symptoms were scored as ‘present’ in case the specific BS item was scored ≥1. For the current bleeding phenotype, the patients reported bleeding requiring treatment in the year prior to inclusion in the study in the questionnaires. Lastly, type 2 and type 3 VWD, RBDs with factor activity below 0.10 IU/mL, Glanzmann thrombasthenia and Bernard Soulier Syndrome were defined as severe bleeding disorders.

### 2.5 Statistical analysis

Continuous data are presented as mean ±standard deviation, whereas categorical data are presented as frequency and proportion (%). Normality of data was assessed visually. Missing data were not imputed.

An independent *t*-test was used to investigate a difference in continuous variables between two groups, whereas ANOVA test was used to investigate a difference between three groups or more. Continuous data were adjusted for relevant confounders using linear regression analysis. Difference in diagnostic delay between men and women was adjusted for type of disease and BS without sex-specific bleeding. Outcomes of linear regression analyses are presented as unstandardized β and 95% confidence interval (CI). Categorical data were compared between two or more groups using a chi-square test. These outcomes were adjusted for type of disease and BS without sex-specific bleeding using logistic regression analysis. Outcomes of logistic regression analyses are presented as odds ratio (OR) and 95% CI.

Time to first bleeding, time to diagnosis and time from first bleeding until diagnosis were compared between men and women with Kaplan–Meier Curves. Based on the event-curves, we used the log-rank test to compare the time to first bleeding between men and women, whereas the Breslow test was used to compare time to diagnosis and time from first bleeding until diagnosis between men and women. A *p*-value below 0.05 was considered to be statistically significant. All analyses were performed with SPSS version 25 (IBM Corp., Armonk, NY, USA).

### 2.6 Role of funding source

The funders did not have any role in design of the study, in data collection, in statistical analysis, in the interpretation of data or in writing of the manuscript. The authors were fully responsible for all aspects of this research.

## 3 Results

We included 1092 patients with VWD (*n* = 834), RBDs (*n* = 196) and CPDs (*n* = 62) in this study, of whom 665 (60.9%) were women (Table 1). Men were slightly younger at inclusion in the studies compared to women, respectively 37.1 years ±21.8 vs 41.2 years ±18.2 (*p* = 0.002, Table 1). The baseline characteristics of the total population are presented in Table 1, whereas the characteristics for each disorder including laboratory measurements are presented in Supplemental Table 1.

**Table 1:** Characteristics of the total study population.

	Men *n* = 427	Women *n* = 665	*P*-value
Age at inclusion, years	37.1 ± 21.8	41.2 ± 18.9	0.002
Age first bleeding, years	8.9 ± 13.6	10.6 ± 11.3	0.075
Age at diagnosis, years	16.6 ± 19.6	22.5 ± 18.4	<0.001
Diagnostic delay, years	7.7 ± 16.6	11.6 ± 16.4	0.002
Referred for bleeding, *n* (%)	145/396 (36.6%)	296/618 (47.9%)	0.002
BS	9.7 ± 6.9	11.6 ± 7.2	<0.001
Abnormal BS	341 (84.2%)	495 (80.6%)	0.145
BS without sex-specific bleeding	9.6 ± 6.9	8.8 ± 6.0	0.036
Bleeding requiring treatment in year prior to inclusion, *n* (%)	153/399 (38.3%)	215/623 (34.5%)	0.213

Data are presented as mean ±standard deviation unless otherwise specified. BS bleeding score. Abnormal BS is defined as ≥3 in children, ≥4 in males and ≥6 in females.

### 3.1 Reason for referral

**Fig. 1:**
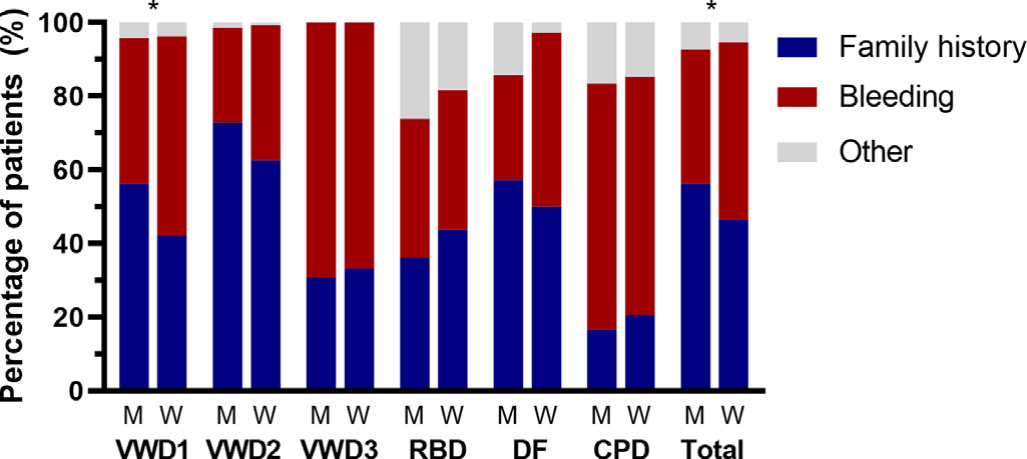
**Reason for referral in men and women with autosomal inherited bleeding disorders**. *p<0.05 between men and women. VWD – von Willebrand disease, RCD – rare coagulation factor deficiencies, DF – disorders of fibrinolysis, CPD – congenital platelet defects.

Of the 1014 patients in whom the reason for referral was known, the most common reason was a positive family history in 510 patients (50.3%), followed by a personal bleeding tendency in 441 patients (43.5%) and other reasons (mostly prolonged prothrombin time (PT), activated partial thromboplastin time (APTT) or platelet function analyzer (PFA) at pre-operative screening) in 63 patients (6.2%). Overall, women were more often referred because of bleeding symptoms than men, respectively 296 of 618 women (47.9%) vs 145 of 396 men (36.6%) (*p* = 0.002) (Fig. 1). Men were more frequently referred because of a family history of bleeding disorders (Fig. 1).

In 399 patients diagnosed with a severe bleeding disorder (i.e. type 2 and type 3 VWD, RBDs with factor activity below 0.10 IU/mL, Glanzmann thrombasthenia and Bernard Soulier Syndrome) of which 192 (48.1%) were men and 207 (51.9%) women, there was no difference in the reason for referral between men and women (*p* = 0.215).

### 3.2 Age of first bleeding and diagnosis

Overall, age of first bleeding was 8.9 ± 13.6 years in men and 10.6 ± 11.3 years in women (*p* = 0.075), whereas age of diagnosis was 16.6 ± 19.6 years in men, and 22.5 ± 18.4 years in women (*p*<0.001, Table 1). Therefore, the diagnostic delay (i.e. time from first bleeding until diagnosis) was markedly longer in women than in men, respectively 11.6 ± 16.4 years vs 7.7 ± 16.6 years (*p* = 0.002). This difference persisted after adjustment for type of disease and BS minus sex-specific bleeding with a difference of β=3.6 (1.1; 6.1) years.

The age of first bleeding was not different between patients who were referred because of a positive family history and patients referred because of bleeding, respectively 8.7 ± 11.9 years vs 10.2 ± 11.8 years, *p* = 0.108. Nevertheless, there was a major difference in age of diagnosis between patients referred because of a positive family history and patients referred because of bleeding, respectively 15.7 ± 17.8 years vs 24.0 ± 18.7 years, *p*<0.001.

**Fig. 2:**
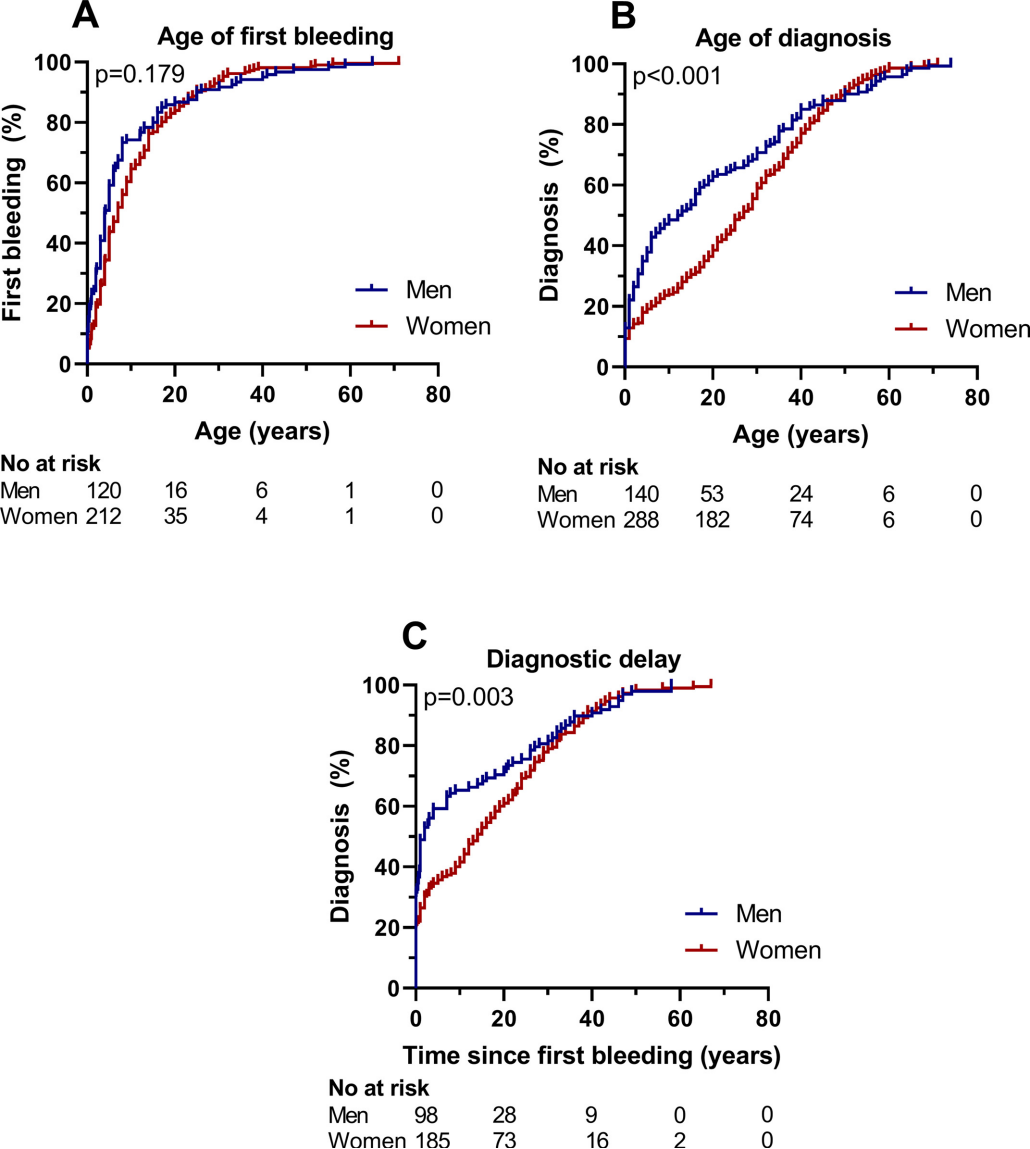
**(A) Time to first bleeding, (B) time to diagnosis and (C) diagnostic delay in patients referred for bleeding**. Data presented as outcomes of Kaplan–Meier curves. (A) Log-rank test was used to compare the time to first bleeding between men and women, whereas Breslow test was used to compare (B) time to diagnosis and (C) time from first bleeding until diagnosis between men and women. No at risk numbers at risk.

In patients referred for bleeding, age of first bleeding was similar between men and women (Fig. 2A, *p* = 0.179), which was on average 9.4 ± 13.0 years in men and 10.7 ± 11.1 years in women (*p* = 0.303). Nevertheless, men were at a much younger age diagnosed than women, especially in childhood (Fig. 2B, *p*<0.001). The average age of diagnosis was 19.4 ± 20.0 years in men and 26.3 ± 17.7 years in women (*p* = 0.001). The diagnostic delay in patients referred for bleeding was longer in women (Fig. 2C, *p* = 0.003). Half of all men referred for bleeding were diagnosed within 2.0 (0.8–3.2) years after their first bleeding, whereas in women this was after 14.0 (10.4–17.6) years (Fig. 2C). The average diagnostic delay was 9.1 ± 17.5 years in men and 14.5 ± 16.5 years in women (*p* = 0.006). After adjustment for type of disease and BS minus sex-specific bleeding, the average diagnostic delay was still longer in women compared to men with a difference of β=5.8 (1.9; 9.7) years. Even in patients with severe bleeding disorders referred for bleeding (*n* = 134), the diagnostic delay was longer in women (10.0 ± 15.4 years) than in men (4.3 ± 14.7 years), *p* = 0.048.

### 3.3 Bleeding during life time

**Fig. 3:**
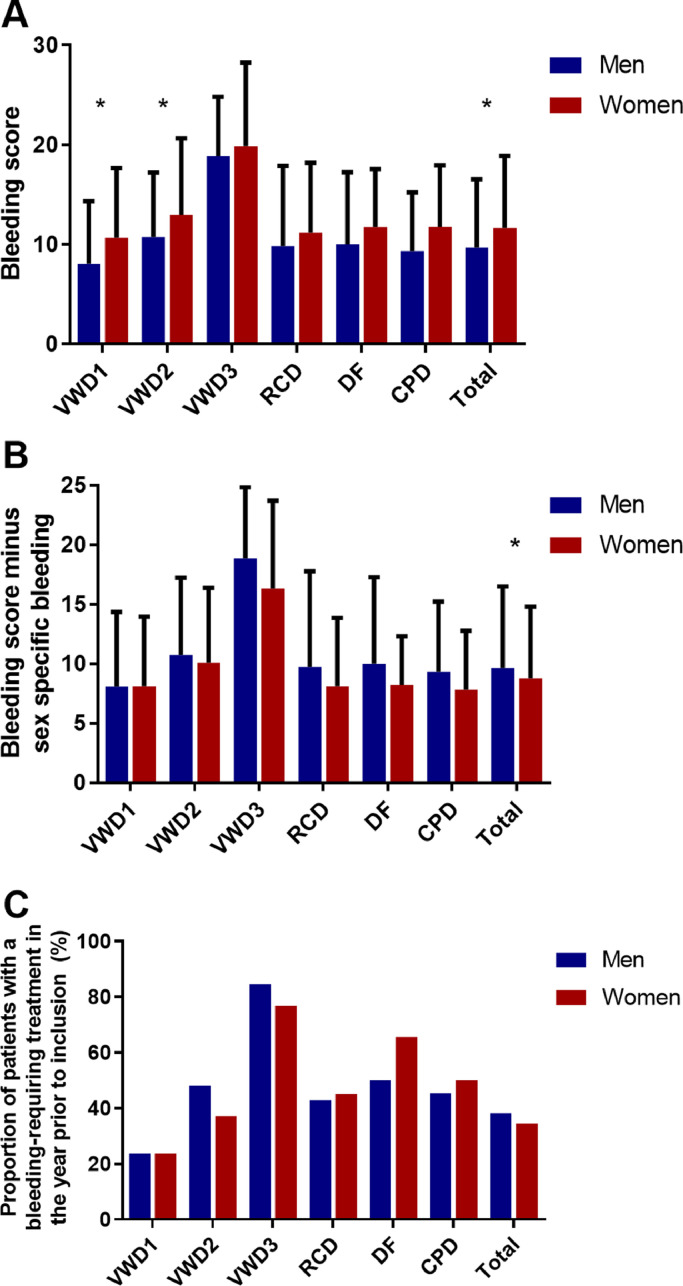
**Bleeding phenotype of men and women with autosomal inherited bleeding disorders**. (A) In the total population, BS was higher women than men, whereas (B) BS minus sex specific bleeding was higher in men than women. (C) No difference was found in percentage of patients with bleeding requiring treatment in the year prior to inclusion in the study between men and women. *p<0.05 between men and women. VWD – von Willebrand disease, RCD – rare coagulation factor deficiencies, DF – disorders of fibrinolysis, CPD – congenital platelet defects.

The BS, which gives an indication of the severity of bleeding episodes during life time, was lower in men (9.7 ± 6.9) compared to women (11.6 ± 7.2, *p*<0.001, Fig. 3A). However, after exclusion of sex-specific bleeding, BS was higher in men than in women, respectively 9.6 ± 6.8 vs 8.8 ± 6.0, *p* = 0.036 (Fig. 3B). After adjustment for age and type of disease, BS minus sex-specific bleeding was still higher in men compared to women with a difference of β=1.2 (0.4; 1.9) points.

### 3.4 Current bleeding phenotype (bleeding symptoms in the year prior to inclusion)

Overall, 153 of 399 (38.3%) men and 215 of 623 (34.5%) women had a bleeding episode requiring hemostatic treatment in the year prior to inclusion in the study (*p* = 0.213) (Fig. 3C). In adult patients, there was no difference in bleeding requiring treatment in the year prior to inclusion between men and women, respectively 112 (36.8%) vs 190 (35.1%) (*p* = 0.616). In children (<18 years), boys seemed to have more often bleeding requiring treatment in the year prior to inclusion compared to girls, although not statistically significant, respectively 41 boys (43.2%) vs 24 girls (29.6%) (*p* = 0.064). After adjustment for age and type of disease, boys seemed to have almost a two times higher chance for bleeding requiring treatment in the year prior to inclusion in the study compared to girls: OR=1.9 (1.0; 3.6).

### 3.5 Sex-specific bleeding and management

HMB was the most common bleeding symptom in women with a prevalence of 81.7% (497 of 608) in women aging 12 years or older. Four hundred-thirty-six women (71.5%) had been treated because of HMB. Ninety-five women (16.7%) had undergone an endometrial ablation or curettage, whereas 66 (11.6%) had undergone a hysterectomy because of HMB. A comparable number of women had used desmopressin, factor concentrates or needed blood transfusion because of HMB, respectively 38 (6.7%), 34 (6.0%) and 40 (7.0%) women. Lastly, 236 women (41.5%) had received iron supplements because of iron deficiency due to HMB.

Of 409 women who had at least one delivery, 204 women (49.9%) reported to have had PPH and 180 (43.8%) had been treated because of PPH. A large proportion of women who had a delivery received a blood transfusion: 79 (20.4%). Additional uterotonic medication, factor concentrates and desmopressin were given in respectively 60 (15.5%), 54 (14.0%) and 16 (4.1%) women with PPH. Four women had to undergo a hysterectomy and five women were treated with antifibrinolytics because of PPH.

Overall, of all women aging 12 years or older, 469 (77.1%) women had received treatment because of sex-specific bleeding, such as HMB and PPH.

## 4 Discussion

In this large combined nationwide study of patients with autosomal inherited bleedings disorders including VWD, RBD and CPD patients, we found major sex differences in the clinical phenotype of patients. Firstly, women were more often referred for bleeding, whereas men were referred more often because of a positive family history. Secondly, the diagnostic delay, defined as time between first bleeding and diagnosis of bleeding disorder, was longer in women than in men, irrespective of the type of bleeding disorder and severity of the bleeding phenotype. Thirdly, due to sex-specific bleeding (HMB and PPH), women had a higher BS than men. However, when sex-specific bleeding symptoms were excluded, men had a more severe bleeding phenotype. In children, boys more often had a bleeding requiring treatment in the year prior to inclusion in the study than girls, whereas in adults there was no difference between men and women.

Remarkably, the diagnostic delay was longer in women with bleeding disorders than in men, independent of the type of bleeding disorder and severity of bleeding phenotype. This is probably because women may have less often traumatic bleeding episodes at early childhood than men. Men may present more often with spontaneous or traumatic bleeding at a younger age, and since these bleeding are not physiologic, they may be investigated early to diagnose or to rule out an underlying bleeding disorder. In accordance, it has previously been shown in the WiN study that boys with VWD are overrepresented at childhood compared to girls with VWD (60% vs 40%), whereas in adults women are overrepresented (64% women vs 36% men) [14,23]. A recent study in children with VWD also found that boys were diagnosed at a younger age and had their first bleed at a younger age than girls with VWD [24]. This study also found that boys with VWD more often had a bleeding and more treatment product use than girls with VWD [24]. Similarly, in our current study, in the total group of autosomal inherited bleeding disorders, boys seemed to have an almost two times higher chance of bleeding requiring treatment in the year prior to inclusion than girls. This is probably due to the fact that boys are more physically active and participate more often in sports with a higher chance of bleeding, as previously shown in VWD patients [[25], [26], [27]]. Women on the other hand, may present more often with HMB as the first symptom of their bleeding disorder. Since oral contraceptive therapy is often used as initial and effective treatment to alleviate menstrual blood loss, this presenting symptom may not be recognized as a first sign of a bleeding disorder, and therefore no laboratory investigation will be performed. About two decades ago, Ragni et al. investigated 38 women with type 1 VWD and Kirtava et al. investigated 75 type 1 VWD women, and found a diagnostic delay of respectively 4 and 16 years in their studies [28,29]. In both studies, most women presented with HMB as first bleeding symptom [28,29]. Other studies have also shown that HMB is often a presenting symptom of a bleeding disorder [11,30,31]. In addition, physicians may consider the presence of a bleeding disorder more often in boys than girls, because hemophilia A and B, the most known bleeding disorders, mainly affect men. Another explanation for the longer diagnostic delay in women is that women with a mild bleeding phenotype are due to menstruation and delivery exposed to physiologic hemostatic challenges throughout life, and can therefore be diagnosed with a bleeding disorder. Men with a mild bleeding phenotype on the other hand, do not have physiologic hemostatic challenges, and may therefore never be diagnosed with a bleeding disorder. Notwithstanding, the diagnostic delay was on average about 6 years longer in women compared to men in our current study, irrespective of the type of disease and the severity of bleeding phenotype. Moreover, half of all men referred for bleeding were diagnosed within 2 years after their first bleeding, whereas in women this was after 14 years. Future studies should investigate patients with bleeding disorders on an individual basis, to identify additional risk factors associated with the diagnostic delay.

This study indicates that there is much to win in diagnosing women with bleeding disorders earlier. When the diagnosis of a bleeding disorder is delayed, women are at risk for not receiving appropriate treatment in case of bleeding or prophylaxis for surgery, dental procedures or during child delivery. In addition, there may be a negative impact on quality of life due to absence at school or work and potentially missing out on social activities and sports [18,32]. Therefore, physicians should be more aware of bleeding disorders in women who present with a personal bleeding diathesis, and should investigate them earlier for bleeding disorders. We recommend general practitioners and gynecologists to ask about other bleeding symptoms and family history of bleeding symptoms and disorders in women presenting with HMB. A formal BS assessment may not be sufficient in such a setting, since in many BS items the points are scored based on whether patients have had treatment for a certain bleeding, and therefore in young women this may yield false negative BS results. Thus, in such women easy bruising, recurrent epistaxis or prolonged bleeding after minor wounds may already be considered as clues for a possible bleeding disorder. In addition, it may be useful to screen family members of index patients to diagnose them earlier.

Furthermore, women were more often referred because of a personal bleeding diathesis than men. This is probably also because women have more physiological hemostatic challenges than men. Therefore, they have a higher chance of experiencing bleeding episodes, and higher chance for referral to diagnose or rule out a bleeding disorder. This partly explains why in our study there were more women with generally milder bleeding disorders (type 1 VWD, mild rare coagulation factor deficiencies, disorders of fibrinolysis and CPDs), whereas the number of women and men with severe bleeding disorders (type 2 and 3 VWD and severe rare coagulation factor deficiencies) were comparable. Similarly, other large studies in patients with VWD and low VWF identified more women than men [9,10]. Because these bleeding disorders have an autosomal inheritance pattern, it seems that a large proportion of men with bleeding disorders with a clinically mild bleeding phenotype are never diagnosed.

HMB was the most common symptom (82%) in women aged 12 years and above, and necessitating treatment in most of these women. In addition, half of all women with a delivery in the past in our study had a PPH. For VWD we have previously reported that 81% of all women experienced menorrhagia (self-reported), whereas over 50% of women experienced bleeding complications after childbirth or pregnancy loss [12]. A previous review on patients with RBDs found that more than 30% of women with RBDs experienced menorrhagia, whereas PPH incidence was uncommon in this population [4]. In CPD, HMB was present in 61% of women, whereas PPH was present in 78% of women who had a delivery [33]. Overall, these results indicate that sex-specific bleeding is an important issue in women with bleeding disorders, and often requires treatment.

This is the first study to investigate sex differences in a large cohort of well-defined patients with different types of autosomal inherited bleeding disorders. We included patients from three nationwide studies in the Netherlands, which is therefore representative for all patients with these disorders in our country. Moreover, the methods of the three large included nationwide studies were very comparable, making the results suitable for combining them.

Nevertheless, there are some potential limitations. Firstly, data on age of diagnosis and age of first bleeding were self-reported. Therefore, this could lead to non-differential information bias and may cause a dilution of effect (i.e. loss of power). Since we found clear differences between men and women, this potential information bias seems negligible, and does not explain the objectified differences between men and women. Secondly, we included more VWD patients than patients with other disorders. However, this represents the prevalence of VWD compared to other bleeding disorders in the general population. Lastly, we acknowledge that future studies should investigate patients with bleeding disorders in an individual basis, to identify other factors associated with the diagnostic delay.

To conclude, there are important differences in presentation, diagnosis and management of men and women with autosomal inherited bleeding disorders. Women are referred more often with bleeding episodes, the diagnostic delay is longer in women, and they often require treatment for sex-specific bleeding. It is of utmost importance that girls and women presenting with bleeding symptoms should be referred and investigated sooner for a bleeding disorder and to extend family investigation to women in case of an established bleeding disorder.

## Data sharing

Original data can be obtained by sending an email to the corresponding author (Frank W.G. Leebeek; f.leebeek@erasmusmc.nl).

## Declaration of Interests

*F. Atiq* received the CSL Behring-professor Heimburger Award 2018 and a travel grant from Sobi. *J.L. Saes* and *S.E.M. Schols* received travel support from Bayer, Takeda and Sobi. *F.W.G. Leebeek* received research support from CSL Behring and Shire/Takeda for performing the Willebrand in the Netherlands (WiN) study, and from UniQure and Sobi for other studies. He is consultant for UniQure, Novo Nordisk, BioMarin and Shire/Takeda, of which the fees go to the institution, and has received a travel grant from Sobi. He is also a DSMB member for a study by Roche. *H.C.J. Eikenboom* received research support from CSL Behring and he has been a teacher on educational activities of Roche. *K.P.M. van Galen* received unrestricted research support from CSL Behring and Bayer and speakers fee from Takeda, CSL Behring and Bayer. *J.G. van der Bom* has been a teacher on educational activities of Bayer. *M.H. Cnossen* has received grants from governmental research institutes, such as the Dutch Research institute (NOW), ZonMW, Innovation fund, NWO-NWA and unrestricted investigator initiated research grants as well as educational and travel funding from various companies over the years (Pfizer, Baxter/Baxalta/Shire, Bayer Schering Pharma, CSL Behring, Sobi Biogen, novo Nordisk, Novartis and Nordic Pharma), and has served as a member on steering boards of Roche and Bayer. All grants, awards and fees go to the Erasmus MC as an institution. The institution of *K. Fijnvandraat* has received unrestricted research grants from CSL Behring, Sobi and NovoNordisk and her institution received consultancy fees from Grifols, Takeda, Novo Nordisk and Roche. *K. Meijer* received research support from Bayer, Sanquin and Pfizer; speaker fees from Bayer, Sanquin, Boehringer Ingelheim, BMS and Aspen; consulting fees from Uniqure, of which all fees go to the institution. *B.A.P. Laros‐van Gorkom* has received unrestricted educational grants from Baxter and CSL Behring. *M.J.H.A. Kruip* received grants from governmental research institutes, such as the Dutch Research institute (ZonMW/NWO), Dutch Thrombosis Foundation, Innovation fund, unrestricted grants from Bayer, Pfizer, Daiichi Sankyo, Sobi and Boehringer Ingelheim and speakers fee from Bayer. *R.E.G. Schutgens* received grants from Bayer, grants from Baxalta, Pfizer, NovoNordisk. The institution of *M. Peters* received an unrestricted research grant from Pfizer. *F.J.M. van der Meer* received grants from CSL Behring, Pfizer, Bayer, Novo Nordisk, Sobi, Roche, and OctaPharma. *W.L. van Heerde* reports speaker and consultant and travel fees from Takeda, Bayer, CSL Behring, and Sobi. He is also founder and co-owner of Enzyre. None of the other authors has a conflict of interest to declare.

## Appendix Supplementary materials

Image, application 1

Image, application 2
