# Supplementary material for: Major differences in clinical presentation, diagnosis and management of men and women with autosomal inherited bleeding disorders
Source: eClinicalMedicine. 2021 Jan 29;32:100726. doi: 10.1016/j.eclinm.2021.100726 (PMC7848767; doi:10.1016/j.eclinm.2021.100726)
Supplement: Supplementary file 1 [file mmc1.docx]

| **Supplemental Table 1. Patient characteristics and baseline laboratory values for each bleeding disorder** | | | | | | | | | | | | | |
| --- | --- | --- | --- | --- | --- | --- | --- | --- | --- | --- | --- | --- | --- |
|  |  | **Type 1 VWD** | | **Type 2 VWD** | | **Type 3 VWD** | | **Rare bleeding disorders** | | **Disorders of fibrinolysis** | | **Hereditary platelet defects** | |
|  | **Reference range** | **Men** | **Women** | **Men** | **Women** | **Men** | **Women** | **Men** | **Women** | **Men** | **Women** | **Men** | **Women** |
| Patients, n (%) |  | 173 (35%) | 321 (65%) | 148 (47%) | 164 (53%) | 14 (50%) | 14 (50%) | 63 (42%) | 88 (58%) | 7 (16%) | 37 (84%) | 22 (36%) | 40 (65%) |
| Age inclusion, years |  | 33.9 ±20.9* | 43.1 ±17.9* | 36.4 ±21.6 | 38.9 ±20.9 | 30.7 ±23.0 | 28.7 ±21.2 | 43.2 ±23.8 | 36.6 ±18.0 | 55.6 ±17.7 | 48.5 ±18.8 | 48.2 ±15.3 | 43.0 ±15.1 |
| Blood group O, n (%) |  | 105 (69%) | 199 (69%) | 52 (42%)* | 80 (56%)* | 5 (35.7%) | 6 (43%) |  |  |  |  |  |  |
| VWF:Ag, IU/mL |  | 0.35 ±0.24 | 0.40 ±0.22 | 0.27 ±0.16 | 0.27 ±0.15 | 0.01 ±0.01 | 0.01 ±0.01 |  |  |  |  |  |  |
| VWF:CB, IU/mL |  | 0.41 ±0.31 | 0.47 ±0.29 | 0.12 ±0.20 | 0.13 ±0.12 | 0.01 ±0.02 | 0.00 ±0.00 |  |  |  |  |  |  |
| VWF:Ab, IU/mL |  | 0.44 ±0.34 | 0.49 ±0.32 | 0.14 ±0.24 | 0.12 ±0.12 | 0.00 ±0.01 | 0.00 ±0.00 |  |  |  |  |  |  |
| FVIII:C, IU/mL |  | 0.62 ±0.33* | 0.70 ±0.31* | 0.37 ±0.17 | 0.42 ±0.27 | 0.06 ±0.14 | 0.02 ±0.01 |  |  |  |  |  |  |
| Platelet count ,*10^9^/L | 150-400 |  |  |  |  |  |  |  |  |  |  | 172 ±106 | 223 ±102 |
| Coagulation factor activity, IU/mL | 0.7-1.2 |  |  |  |  |  |  | 0.23 ±0.28 | 0.24 ±0.30 |  |  |  |  |
| Fibrinogen activity, g/L | 1.6-3.2 |  |  |  |  |  |  | 0.46 ±0.26 | 0.70 ±0.40 |  |  |  |  |
| PAI-1 antigen levels, ng/mL | 3.4-39 |  |  |  |  |  |  |  |  | - | 2.3 ±0.6 |  |  |
| A2-AP levels, U/mL | 87-114 |  |  |  |  |  |  |  |  | 0.45 ±0.30 | 0.39 ±0.45 |  |  |
| Eugbolulin clot lysis time ratio | >5.6 |  |  |  |  |  |  |  |  | 11.0 ±2.4 | 8.1 ±2.8 |  |  |
| Data are presented as mean ±sd unless otherwise specified. *p<0.05 between men and women. Of patients with RBD, 29 patients had a hypo/a/dysfibrinogenemia, 11 had FII deficiency, 23 had FV deficiency, 3 had combined FV+FVIII deficiency, 44 had FVII deficiency, 5 had FX deficiency, 28 FXI deficiency and 9 FXIII deficiency. Of patients with a disorder of fibrinolysis, 20 patients had hetero/homozygous α2-antiplasmin deficiency, 11 had PAI-1 deficiency and 13 had unclassified hyperfibrinolysis. Of the CPD patients, 4 had Bernard Soulier Syndrome, 14 Glanzmann thrombasthenia, 17 ADP pathway defect, 14 TxA2 pathway defect and 13 Dense granule deficiency. | | | | | | | | | | | | | |
